# Living with type 2 diabetes: stress and daily life challenges among adults in the Ashanti Region, Ghana

**DOI:** 10.1186/s12889-026-28131-4

**Published:** 2026-06-12

**Authors:** Godfred Darko, Peter Sadari, Daniel Quarshie, Georgina Anokye Agyekum, Yula Salifu, Frank Offei Odonkor, Kofi Sekyere Boateng

**Affiliations:** 1 Department of Public Health Education, Faculty of Environment and Health Education, Akenten Appiah-Menka University of Skills Training and Entrepreneurial Development, Kumasi, Ghana; 2 Department of Nursing, Faculty of Nursing and Midwifery, University for Development Studies, Tamale, Ghana; 3 Department of Population, Family and Reproductive Health, School of Public Health, University of Ghana, Legon, Ghana; 4 Department of Adult Health, School of Nursing and Midwifery, University of Cape Coast, Cape Coast, Ghana

**Keywords:** Diabetes distress, Type 2 diabetes, Taboos, Transport barriers, Occupational stress, Ghana

## Abstract

**Background:**

Diabetes distress drives treatment non-adherence and poor quality of life among Ghanaian adults with type 2 diabetes. Terrain, occupational, and Akan cultural stressors in high-burden peri-urban communities remain underexplored.

**Objective:**

This study examined lived experiences of stress, sociocultural barriers, and impacts on daily quality of life among adults with type 2 diabetes across agrarian (Mampong Ashanti) and mining (Obuasi) communities in Ghana’s Ashanti Region.

**Methods:**

We conducted a descriptive qualitative study with purposive sampling of 92 adults (30–45 and > 45 years) via 16 FGDs (6–10/group) and 12 IDIs with health workers (6/district) in two districts. Bilingual guides explored stress triggers, coping, taboos, and WHOQOL domains (Nov-Dec 2025). NVivo 14 content analysis included triangulation, member checking, and interrater reliability (≥ 85).

**Results:**

Five themes emerged with ≥ 85% source convergence across 92 patients and 12 providers: medication poverty, transport barriers, occupational conflicts, fears of complications, and family-related stigma. Medication poverty was reported across all focus groups, while transport barriers were particularly prominent among participants in Mampong. Occupational conflicts were uniquely emphasized by miners in Obuasi. Older adults frequently expressed fears of complications, whereas younger participants highlighted experiences of stigma within family and social contexts. Additionally, Akan cultural taboos were described as restricting shrine access, church participation, and communal feasts during hyperglycaemic episodes.

**Conclusions:**

Diabetes distress in the Ashanti Region is multifactorial, influenced by geography, occupation, generational differences, and cultural norms. Interventions should integrate psychosocial screening, medication affordability, CHPS satellite clinics, culturally sensitive peer support, and engagement with traditional healers to improve adherence and quality of life.

**Supplementary Information:**

The online version contains supplementary material available at 10.1186/s12889-026-28131-4.

## Introduction

Stress management among adults with diabetes is critical to achieving the Sustainable Development Goals (SDGs) [1], mainly SDG 3 (good health and well-being), SDG 8 (decent work and economic growth), and SDG 10 (reduced inequalities), as outlined in the United Nations 2030 Agenda for Sustainable Development and subsequent SDG progress reports [2–4]. These goals are essential for addressing the needs of diabetic patients, especially those in vulnerable situations within low-resource settings [5]. Adults living with diabetes face multiple challenges managing stress at home, workplaces, and clinics [6]. These challenges are compounded by factors such as medication costs consuming 15–25% of household income, transport barriers to clinics, and inadequate counseling facilities, especially in peri-urban areas [7]. Other key barriers include diabetes stigma and taboos, treatment fatigue, peripheral neuropathy pain, and associated symptoms such as anxiety, hypoglycaemic distress, fatigue, and complication fears [7–9]. The quality of life and dignity of diabetic adults in low-income settings are undermined by these persistent stress-related challenges [10].

Moreover, many patients lack adequate knowledge about stress-diabetes connections and information on effective coping practices [11]. This stems partly from cultural silences surrounding chronic illness. For instance, diabetes stigma manifests universally across Asia, Africa, Europe, and America [12, 13]. In some communities like rural India and Haiti, symptomatic patients face isolation periods resembling quarantine [14, 15]. Among Akans in Ghana’s Ashanti Region, diabetics encounter restrictions including prohibitions against shrine visits, communal feasts, and spousal relations during hyperglycaemic episodes [16]. These practices underscore persistent social exclusion of diabetics during symptomatic periods. The 2021 Ghana Population and Housing Census indicates that alongside Christianity and Islam, a proportion of residents in the Ashanti Region still identify with indigenous religious beliefs, which reflects the continuing influence of Akan cosmological systems in everyday health perceptions and behaviours [17, 18].

At times, medically advised rest during flares may feel beneficial [19]; however, such restrictions often reinforce dependency stereotypes [20]. Urgent attention is needed to address diabetes stress challenges including stigma and taboos (and the fears they generate), poor coping practices, and the lack of effective strategies for symptom management [21]. Stress constraints contribute to “diabetes poverty” compounded by inadequate education and shaming over visible symptoms like fatigue or amputations [22, 23]. This restricts socio-economic participation among adults in low- and middle-income countries [24, 25]. Addressing these concerns will promote healthier, more productive lifestyles while safeguarding patients’ dignity [26]. Recent diabetes campaigns emphasize positive self-management and empowerment [27]. Efforts to change narratives around diabetes stress drive interventions providing relevant knowledge, skills, and peer support [28]. Such interventions create opportunities for patients, families, and communities to openly discuss diabetes myths, often intrinsically linked to local cultural taboos [29, 30].

Ghana Health Service’s NCD Strategic Plan demonstrates policy attention to diabetes management [31, 32]. However, these policies remain largely clinic-focused, with limited emphasis on community psychosocial interventions, particularly in Ashanti Region where poverty, occupational hazards, and cultural barriers disproportionately affect diabetic adults [31, 33]. Peer-led, community-driven models have demonstrated effectiveness in other chronic disease contexts, such as HIV care [34], yet they remain rarely integrated into diabetes care in Ghana.

Consequently, the literature remains fragmented, with limited qualitative evidence that systematically weaves together psychosocial pressures, sociocultural norms, occupational constraints, generational differences, and health-system barriers within a comparative, community-based framework. Our study responds to this gap by examining the lived experiences of adults with type 2 diabetes across two contrasting settings in Ghana’s Ashanti Region: (1) the agrarian communities of Mampong Ashanti and (2) the mining communities of Obuasi.

## Materials and methods

Reporting follows COREQ guidelines [35, 36]; completed checklist in Supplementary File 2.

### Study area

Data collection spanned Mampong Ashanti Municipal and Obuasi Municipal Assemblies within Ghana’s Ashanti Region. These two districts were strategically selected for their documented elevated diabetes prevalence (8–12% vs. national 8.3%) [37], complementary demographic profiles enabling rural-peri-urban contrasts, and established clinic infrastructure supporting participant recruitment. Mampong Ashanti Municipal (estimated population 149,210) encompasses peri-urban agricultural communities dominated by Akan ethnicity (87.4%) and Christianity (72.1%) [38], where Mampong Municipal Hospital serves as primary/tertiary referral hub for an expansive hilly catchment area spanning 40 km radius. Geographic challenges including steep gradients, unpaved access roads during rainy seasons, and transport costs (GHS 15–25 per trip/ USD 1.00-1.70) systematically hinder clinic attendance among low-income farming households [39].

Obuasi Municipal (estimated population 225,000; [40]), by contrast, integrates dense mining-industry hubs serviced by AngloGold Ashanti medical facilities alongside sprawling rural agricultural fringes (Akan 82.3%, mixed Christian/Muslim faiths). Occupational exposures including shift work disrupting medication timing, silica dust impairing pulmonary function [41], and elevated BMI from canteen obesogenicity amplify cardiometabolic risks, yielding diabetes prevalence 10–12% in mine-adjacent communities [42]. Both districts exceed national prevalence estimates, offering complementary insights into agrarian versus industrial stressors and supporting a dual-site purposive design over a single-center study.

### Study design and sampling

This qualitative descriptive study employed content analysis to examine lived stress experiences and quality-of-life dynamics among adults with type 2 diabetes and their healthcare providers. Purposive sampling systematically prioritized adults diagnosed ≥ 1 year, aged 30–65 years, actively engaged in care at Mampong Municipal Hospital and Obuasi Government Hospital registries, and diverse health workers (nurses, physicians, counselors). This explicitly favored rich experiential depth and contextual availability over probabilistic representativeness consistent with phenomenological inquiry principles [43]. Participants were stratified into two age groups (30–45 years and > 45 years) to capture potential differences in diabetes stress experiences related to life stage, occupational responsibilities, and generational perceptions of complication fears and stigma. This strategy yielded theoretically homogeneous yet demographically varied rural-peri-urban voices capturing Akan cultural inflections alongside occupational diversity.

### Data collection

Fieldwork spanned 22nd November through 11th December 2025 across eight communities (four per district, aligned with high-burden clinic catchments). Data collection comprised 16 Focus Group Discussions (FGDs) constituting eight homogeneous patient groups (two per district × four clinics: adults > 45 years and 30–45 years; 6–10 participants each; total *n* = 92 patients) plus 12 In-Depth Interviews (IDIs) with providers (six per district: two nurses, two physicians, two counselors). Recruitment proceeded through clinic provider referrals targeting local residents diagnosed during study period, stratified by age/gender/disease duration, explicitly excluding facility staff to minimize power differentials. All invited participants agreed to participate; no refusals or dropouts were reported.

Semi-structured interview guides were developed to operationalize key domains including stress triggers (financial, relational, and clinical), coping strategies, and sociocultural barriers and facilitators. Quality of life was explored using domains informed by the World Health Organization Quality of Life framework (WHOQOL-BREF), which covered physical health, psychological health, environmental, and social relationships [44]. Data collection was conducted by trained bilingual facilitator-note taker pairs (mixed gender: D.G., male; G.A.A., female) fluent in Asante Twi and English. English was used as the primary language of interviews, while Asante Twi was used selectively to clarify participant responses and ensure accurate understanding where necessary. In Obuasi, additional mine-worker translators were engaged to ensure accurate interpretation of mining-related and occupational terminology.

FGDs/IDIs averaged 45–60 min, which yielded verbatim audio recordings. Group sizes of 6–10 participants were used to facilitate rich shared narratives while balancing data saturation considerations and logistical constraints within rural clinic schedules.

### Ethical considerations

All study protocols for this study received ethical clearance from the Ghana Health Service Ethics Review Committee (GHS-ERC: 053/09/25). Procedures included verbal and written informed consent, with witnessed consent for participants with low literacy and administration in local dialect where necessary. Risk was minimized through voluntary participation, flexible scheduling, and the use of community-appropriate venues. Strict confidentiality, anonymity, and participants’ rights were maintained throughout the study.

### Data management and analysis

FGD/IDI audio files underwent rigorous three-phase transcription protocol; (1) initial verbatim capture integrating field notes, (2) bilingual verification against originals (English) by independent transcribers. Although, back-translation was not performed, translation accuracy was ensured through independent bilingual review and consensus checking, (3) researcher audit cross-checking 20% random sample [45], before systematic import into Excel for open coding and NVivo 14 for data management and coding organization.

Data were analyzed using a structured qualitative content analysis approach. Initial coding was guided deductively by pre-defined domains in the interview guide (stress sources, quality of life impacts, and coping strategies), while allowing inductive codes to emerge from participants’ accounts to capture context-specific meanings. Meaning units were identified, condensed, and organized into codes, which were subsequently grouped into categories and developed into final themes [46].

Meaning units were identified through repeated reading of transcripts, where segments of text reflecting a single idea and experience were extracted, condensed, and assigned descriptive codes. Related codes were grouped into categories based on similarities and differences across transcripts. These categories were then reviewed and synthesized into higher-order themes that captured recurring patterns in participants’ experiences across study sites and participant groups.

Coding discrepancies were resolved through discussion and consensus between investigators. Categories were compared across transcripts using a constant comparative approach to identify recurring patterns and ensure consistency across districts, age groups, and occupations [47]. Independent coding by two investigators (GD and SP) achieved ≥ 85% interrater reliability [48]. This was complemented by structured peer debriefing sessions [48], member checking during final FGDs [49], and illustrative verbatim quotations to preserve contextual meaning [50].

An audit trail documenting coding decisions, category development, and theme refinement was maintained throughout the analysis to enhance methodological transparency and trustworthiness.

### Reflexivity

Reflexivity was maintained throughout data collection and analysis by the research team (GD, GAA, SP), composed of public health and nursing researchers. The team acknowledged that prior clinical and academic experiences could influence interpretation of participants’ accounts regarding diabetes stigma, occupational stress, and cultural beliefs.

FGDs and IDIs were conducted using a bilingual approach, with English as the primary language and Asante Twi used for clarification and to ensure participant understanding and comfort. Reflexive field notes were systematically documented during data collection to capture researcher assumptions, emotional responses, and emerging interpretations. These notes were actively reviewed during coding meetings and data interpretation sessions and used to question and refine category development where participant meanings differed across occupational groups.

## Results

### Study participants

Table 1 summarizes the composition of the focus group discussions (FGDs) conducted across eight high-burden diabetes clinics in Mampong Ashanti (*n* = 44 patients) and Obuasi Municipal (*n* = 48 patients). A total of 92 adults with Type 2 diabetes participated in 16 FGDs, complemented by 12 in-depth interviews (IDIs) with health workers (6 per district). The median participant age was 52 years (IQR 38–62), with 54% female representation. Health worker participants comprised 4 nurses, 4 physicians, and 4 counsellors. FGDs were stratified by age (30–45 years; >45 years) and conducted across high-burden clinics in each district.

**Table 1:** Number of participants for the FGDs in the Study

District	Clinic	Age Category	FGD Size	Total
Mampong Ashanti	Mampong Hospital A	30–45 years	8	16
		> 45 years	8	
	Mampong Hospital B	30–45 years	7	15
		> 45 years	8	
	Mampong Health Centre	30–45 years	8	13
		> 45 years	5	
Obuasi Municipal	Obuasi Govt Hospital A	30–45 years	9	17
		> 45 years	8	
	Obuasi Govt Hospital B	30–45 years	8	16
		> 45 years	8	
	AngloGold Clinic	30–45 years	9	15
		> 45 years	6	
Total				92

### Thematic structure

Five core themes emerged from the qualitative content analysis, achieving ≥ 85% source triangulation across FGDs and IDIs, with analytic saturation reached by the 12th FGD (Tables 2 and 3). The themes aligned with established stress typologies.

**Table 2:** Diabetes stress education and existing challenges from focus group discussions

Focus groups contributing to themes	Themes	Mampong Ashanti	Obuasi	> 45 years	30–45 years	Some referenced quotes
Sources of diabetes education	(i) Family and church	8	8	8	8	*“Our wives*,* brothers and from church taught us.”*
	(ii) Clinics/hospitals	8	8	7	8	*“…at clinic our nurse taught us managing blood sugar.”*
	(iii) Others (peers, NGOs, community programs)	4	5	3	6	*“Miner colleagues shared insulin experiences.”*
Existing diabetes stress challenges	(i) Medication costs	8	8	8	8	*“Insulin GHS150 (*USD 10) *but pension only GHS200 (*USD 13) *monthly.”*
	(ii) Transport barriers	8	5	7	6	*“Hilly road to hospital takes 2 h walking.”*
	(iii) Complication fears	7	8	8	7	*“Saw colleague lose leg now I check feet daily.”*
	(iv) Family stigma/support gaps	6	7	5	8	*“Children call me sugar mummy.”*
	(v) Occupational stress	5	8	6	7	*“Night mining shift but must eat fixed times.”*

**Table 3:** Triangulation Matrix Demonstrating Theme Convergence and Saturation

Perspective	Mampong Ashanti (*n* = 44 patients, 6 IDIs)	Obuasi Municipal (*n* = 48 patients, 6 IDIs)	Age 30–45 years (*n* = 46)	Age > 45 years (*n* = 46)	Consistency Across Sources
Patient FGDs (*n* = 92)	Geographic barriers dominant. 8/8 FGDs: Transport costs 7/8 FGDs: Hilly terrain *“2 hrs walk*,* sugar drops halfway”*	Occupational conflicts dominant. 8/8 FGDs: Mining shifts. 7/8 FGDs: Farm timing *“Night shift but must eat 6pm”*	Family dynamics dominant. 8/8 FGDs: Spousal resistance. 7/8 FGDs: Children stigma *“Kids call me sugar mummy”*	Complication fears dominant. 8/8 FGDs: Amputation fears. 7/8 FGDs: Blindness anxiety*” Saw brother lose leg”*	95% theme agreement (FGDs- IDIs)
Health Worker IDIs (*n* = 12)	Transport/non-adherence link 6/6 IDIs: Clinic defaulting *“80% miss due to hills”*	Shift work/glycaemic chaos. 6/6 IDIs: Erratic control *“Miners worst adherence”*	Stigma driving secrecy 7/12 IDIs: Family conflicts *“Young hide from spouses”*	Late complications 8/12 IDIs: Neuropathy dominant *“Elderly present advanced”*	89% FGD-IDI alignment
Prevalence (%)	Transport (100%), Medication costs (88%)	Occupational (100%), Family stigma (88%)	Stigma (94%), Work conflicts (81%)	Complications (100%), Spiritual fears (88%)	All themes ≥ 85% convergence
Saturation	Achieved FGD 7 (no new transport codes)	Achieved FGD 9 (no new occupational codes)	Achieved FGD 8	Achieved FGD 10	Confirmed 12th FGD (final 4 yielded 0 new codes)

The five core themes encompassed:

-  ✓ Structural stressors (medication poverty, transport barriers).
- ✓ Relational stressors (stigma and family dynamics),
- ✓ Occupational stressors (work-regimen conflicts),
- ✓ Clinical stressors (complication fears)
- ✓ Cultural stressors (taboos, spiritual beliefs).

### Theme 1: medication poverty

Medication unaffordability emerged as the most pervasive and immediate stressor across all study sites and participant groups, profoundly affecting both treatment adherence and psychological well-being. Monthly insulin costs, ranging from GHS 150–300 (USD 10–20), often exceeded pension incomes for older adults and represented a substantial fraction of daily wages among farmers, miners, and other low-income earners. As a result, patients frequently experienced treatment interruption, dose skipping, and inconsistent glucose management, creating a cycle of financial strain and health vulnerability.

Participants described a range of coping strategies in response to these economic pressures. These included rationing medication by reducing doses, splitting tablets, alternating injection days, temporarily substituting prescribed medication with herbal remedies, or even delaying refills until financial resources became available. Such adaptations, while pragmatically necessary, often led to unstable glycaemic control and heightened anxiety about potential complications.

Health workers consistently corroborated these observations, emphasizing that non-adherence was rarely due to ignorance or lack of motivation but reflected the realities of economic survival. They noted that patients often understood dosing schedules and the importance of regular medication but were forced to prioritize basic sustenance or family welfare over optimal diabetes management. Clinicians expressed concern that persistent medication poverty could precipitate severe complications, further entrenching the cycle of poverty and disease.

These perspectives are captured in the excerpts below:

*“* *Three days no insulin, I see black and nothing else… Family say witchcraft, not sugar.* *”*

(Mampong, > 45 years)

*“Sometimes I choose food for children or insulin for myself. I cannot do both…”* (Obuasi, 30–45 years).

*“We know the dosage*,* but money decides the dose…”* (Mampong, 30–45 years).

Clinicians observed these constraints:

*“Medication poverty is the biggest driver of non-adherence…. It is not ignorance*,* it is survival economics.”* (Obuasi physician, IDI).

### Theme 2: terrain-specific transport barriers

Geographical access barriers emerged as a significant source of stress, particularly for participants in Mampong Ashanti. The hilly terrain, combined with long travel distances to health facilities, made routine clinic attendance physically demanding and time-consuming. Seasonal road inaccessibility, especially during the rainy season, further complicated travel, often forcing patients to navigate muddy, slippery paths or rely on irregular and costly public transport. Many participants described journeys that involved long uphill walks, waiting for intermittent transport, and coping with extreme heat or rain, leading to fatigue, dizziness, and in some cases, acute hypoglycaemic episodes before reaching the clinic. These challenges often forced patients to prioritize urgent health needs over regular monitoring, promoting reliance on emergency visits rather than scheduled care. The financial burden of transport compounded these difficulties, with patients sometimes skipping visits due to costs, particularly when travel required multiple trips or extended stays away from home.

In contrast, participants from Obuasi reported fewer terrain-related barriers due to relatively flatter urbanized areas and better road networks. However, time-related barriers tied to work schedules, shift patterns, and mining duties were commonly cited. Workers often had to balance strict shift requirements with clinic appointments, creating stress and occasional delays in care.

Health workers corroborated these observations, noting that geographical and temporal barriers in Mampong were strongly linked to missed appointments, inconsistent follow-ups, and delayed care-seeking. They emphasized that these transport-related stressors not only affect adherence to treatment but also exacerbate psychological strain, as patients face both the logistical challenges of travel and the worry about potential deterioration in their health while en-route to care.

These experiences are reflected in participants’ narratives:

*“Two hours walking*,* sugar drop halfway. I nearly collapse*.*”*

(Mampong, 30–45 years)

*“Sometimes you reach clinic already shaking. The journey itself is the sickness.”*

(Mampong, > 45 years)

In contrast, Obuasi participants reported fewer terrain barriers but persistent cost challenges.

Health workers confirmed systematic defaulting:

*“Over 80% of defaulters come from the hills. Distance coupled with fatigue equals non-adherence.”* (Obuasi physician, IDI).

### Theme 3: occupational conflicts

Work-treatment incompatibility emerged as a defining stressor, particularly among Obuasi miners and Mampong farmers, and significantly shaped daily experiences of diabetes management. In Obuasi, miners faced rigid shift schedules that often conflicted with essential self-care routines. Night shifts, extended overtime, and rotating schedules disrupted the timing of insulin injections, blood glucose monitoring, and meal consumption, producing frequent episodes of hypoglycaemia or hyperglycaemia. Miners described situations where the biological demands of their disease were at odds with workplace expectations, forcing them to improvise dosing or skip meals, which compounded stress and anxiety.

In Mampong, farmers encountered unpredictable agricultural cycles, with long days in physically demanding work and seasonal peaks that interfered with timely medication intake and clinic attendance. Some participants described planting, harvesting, or market work taking precedence over healthcare routines, leaving them with little flexibility to manage symptoms or monitor blood sugar. These occupational pressures led many to conceal their condition from supervisors or peers to avoid stigma, job loss, or perceived weakness, creating additional psychological strain.

Representative quotations from participants are presented below:

*“Night shift finish 2am, but must test 6pm. Supervisor no understand diabetes.” *(Obuasi miner, 30–45 years)

“It is hard trying to align my medication with shift duties. During night shifts, I sometimes miss insulin and delay meals because I cannot leave my post. I feel my sugar going up or down, and I know it is because my job does not allow me to manage the condition properly.” (Obuasi miner, > 45 years).

*“Farm work decides my sugar*,* not the doctor.”* (Mampong farmer, > 45 years).

*“If I stop work to eat*,* I lose wages. If I don’t eat*,* sugar drops.”* (Obuasi miner, > 45 years).

Health workers noted erratic glycaemic patterns linked to shift work:

*“Miners have the worst glycaemic stability because their routines are biologically disruptive*.*”* (Health worker, IDI)

### Theme 4: generational complication fears

Generational differences shaped how participants understood and responded to the risk of diabetes-related complications. Older adults expressed fear that were grounded in direct and observed experiences of severe physical deterioration. Younger adults emphasized psychosocial consequences associated with stigma, disclosure, and identity protection.

Among participants aged over 45 years, fear of complications was strongly linked to prior exposure to severe diabetes outcomes within families, communities, and healthcare settings. These experiences influenced daily self-monitoring behaviours and heightened sensitivity to minor bodily changes. Participants described living with persistent awareness of potential limb loss and vision impairment:

“*I remember clearly when my elder brother developed a small wound that never healed. It got worse until he lost his leg at Komfo Anokye Teaching Hospital. Since that time*,* even a small cut on my foot makes me afraid. I check my feet every night before sleeping and in the morning before going to farm work.”* (Mampong, > 45 years).

“*For me*,* it is not even the sugar itself that scares me. It is what it can do. I have seen people become blind and depend on others for everything. That is why I always make sure I take my drugs and avoid anything that will raise my sugar*.” (Obuasi, > 45 years).

These accounts indicate that older adults’ self-management practices were strongly shaped by anticipatory fear of disability. This was further reinforced by observed complications within their social networks.

In contrast, younger adults (30–45 years) described diabetes as a condition deeply tied to social exposure, identity protection, and fear of discrimination. Many participants reported actively concealing their diagnosis in workplace, and social settings to avoid stigma. This concealment shaped both treatment adherence and healthcare-seeking behaviour:

“*The disease itself is not what disturbs me most. What I fear is people finding out. At work*,* if they know*,* they will treat you differently. Even in relationships*,* people start thinking you are cannot support a family. So I take my medicine quietly and avoid testing in public*.” (Obuasi, 30–45 years).

“*Sometimes I hide my medication in food containers when I am at social gatherings. If I do not do that*,* people will start asking questions and spreading rumours. I even miss doses when I am not at home just to avoid attention.”* (Mampong, 30–45 years).

Health workers corroborated these generational differences, noting that older adults were more focused on physical complications, while younger patients often delayed care due to stigma-related concealment:

“*Older patients are mostly worried about what they have seen before*,* and what the disease can do as they grow. Some tell their experiences with relatives who went through complications (blindness*,* amputations*,* stroke). Younger ones are more concerned about who knows their status. Many of them come late because they are trying to hide it from family or partners.*” (Mampong nurse, IDI).

### Theme 5: Akan taboos and stigma

Cultural interpretations of diabetes within Akan cosmology framed hyperglycaemia as a form of ritual impurity, generating exclusionary practices across spiritual, and social domains. From our engagements with participants, they were being restricted from shrine participation, church activities, communal meals, and key social rituals during symptomatic periods, with diabetes interpreted not primarily as a biomedical condition but as a form of spiritual contamination or moral imbalance. These practices were enforced by family members, religious leaders, and community authorities, embedding stigma within everyday moral and religious life rather than isolated individual prejudice. As a result, diabetes management became not only a medical task but also a socially regulated identity, shaped by fear of spiritual defilement, ancestral displeasure, and supernatural attribution.

Workplace stigma further intensified social isolation, particularly in Obuasi, where labeling, ridicule, and symbolic exclusion (avoidance of shared utensils, mockery, and name-calling) led many participants to conceal self-care practices such as glucose testing and medication use. This concealment transformed routine diabetes management into a source of anxiety and secrecy. Gendered stigma also emerged strongly, with women facing narratives around infertility.

These interpretations are illustrated in participants’ own words:

*“Pastor say no church when sugar high… we are unclean.”* (Mampong nurse).

*“No fufu when flare*,* ancestors vex.”* (Obuasi, > 45 years).

*“Shrine priest say sugar is spiritual pollution.”* (Mampong, > 45 years).

Workplace stigma compounded these beliefs:

*“They laugh and call me ‘sugar man’. I hide my testing.”* (Obuasi miner).

A counselor’s account highlighted the profound impact of cultural and religious taboos on clinic attendance:

*“Patients often miss clinic appointments to pursue herbal cleansings. Churches restrict them during flares*,* shrines label it spiritual pollution*,* and families even hide their food. These taboos contribute to around 70% of missed visits*.” *(Obuasi counselor*,* IDI)*.

### Deviant cases

A small number of deviant cases (*n* = 3) were identified that did not contradict the dominant thematic structure. It only demonstrated how outcomes varied when protective factors were present within otherwise similar stress environments.

In two instances, participants reported adherence to treatment despite exposure to the same financial and occupational constraints described across the study. A female participant (Obuasi, 30–45 years) attributed her stability not to reduced stress exposure but to sustained household support and structured reminders, noting: *“Even when money is not enough*,* my family makes sure I don’t forget. They remind me morning and evening*,* so it has become part of my routine.”* This indicates that adherence was maintained through external reinforcement rather than absence of stressors.

Similarly, a male participant (Obuasi, 30–45 years) maintained open disclosure in the workplace without experiencing the concealment patterns commonly reported elsewhere. This was linked to a supportive supervisory environment rather than absence of stigma in the wider setting, as he explained: *“I still hear jokes about diabetes*,* but my supervisor told me to manage my health first. That is why I can step out confidently when needed without fear.”*

Another case (female, Mampong, > 45 years) diverged from the dominant cultural explanatory framework but did so in a way that did not alter her exposure to community norms. Instead, she actively resisted them while remaining within the same social environment: *“People say it is spiritual and sometimes recommend their spiritual pastors*,* but I don’t accept that. I just follow what the clinic says*,* even if others disagree.”*

## Discussion

Our study revealed that the primary sources of diabetes stress education among participants were family members and clinic staff across both age groups in Mampong Ashanti and Obuasi districts. This aligns with prior research highlighting the significant role of spouses and health workers as key conveyors of diabetes knowledge in Ghanaian contexts [51]. However, reliance on these sources appeared limited in explaining the bidirectional relationship between stress and glycaemic control [52], consistent with global diabetes distress literature [53]. Beyond family networks, participants reported support from churches and peers, with clinics providing the most valued technical guidance on insulin administration and foot care. Patients in Mampong reported limited peer support compared to Obuasi miners, likely reflecting occupational solidarity among mine workers versus the relative isolation of farming communities [54]. These findings corroborate evidence that multi-channel education enhances chronic disease management in low-resource settings [50–52].

The main diabetes stress challenges: medication poverty, transport barriers, complication fears, family stigma, and occupational conflicts are consistent with literature on diabetes distress in sub-Saharan Africa [55]. From our findings, Medication costs consumed a substantial proportion of household income, exceeding the WHO/Health Action International affordability benchmark, which defines essential medicines as affordable when the cost does not exceed one day’s wage of the lowest-paid unskilled government worker for a month’s supply. Using Ghana’s estimated unskilled daily wage (about GHS 18–25/ USD 1.20–1.70), monthly insulin costs in this study (GHS 150–300/ USD 10–20), were about 6 to 17 times above the affordability threshold. This substantial cost burden contributes to treatment interruption and non-adherence, consistent with evidence from other chronic disease management studies in low-resource settings [56, 57].

Transport barriers were also consistently emphasized by participants in Mampong and have similarly been associated with increased clinic absenteeism in other hilly regions of Ghana [58]. Fears of complications were particularly salient among older participants, aligning with documented amputation anxiety in African diabetes cohorts [59]. Family stigma manifested as spousal resistance and children’s nicknames, paralleling HIV-related discrimination patterns in Ghana [60, 61]. Obuasi miners uniquely reported shift work conflicts disrupting fixed meal timing, representing a novel occupational stressor absent in prior clinic-based studies [62].

Religio-cultural taboos and stigma persisted across contexts. Shrine exclusions during glycaemic flares resembled culturally documented menstrual restrictions within Akan communities [63, 64], while church prohibitions against altar participation during “sugar highs” reflected spiritual contamination fears analogous to Islamic menstrual exclusions [65]. No major contextual differences were observed between districts regarding these cultural interpretations, suggesting entrenched Akan cosmology transcends Christian/Muslim divides. Participants believed that violations of these taboos contributed to prolonged hyperglycaemia, reinforcing fear through confirmation bias, which reflects culturally embedded explanatory beliefs around bodily states [66]. Workplace stigma among younger miners (“sugar man” taunts) paralleled schoolgirl teasing, although incidents rarely escalated due to supervisor intervention. Health worker triangulation further supported the role of stress in non-adherence, consistent with reported clinic default patterns, lending contextual support to patient narratives through observed patterns of clinic default and reported glycaemic instability [67].

Gender influenced how participants experienced and responded to diabetes-related stress across Themes 3, 4, and 5, although the major stressors were shared across both men and women. Men who worked in mining, often described difficulties managing medication schedules, meals, and glucose monitoring. This was due to long shifts and physically demanding work. Women, on the other hand, frequently described the challenge of balancing diabetes care with caregiving responsibilities, household duties, and informal trading activities. Differences were also evident in perceptions of complications, where women often expressed concern about becoming dependent on family members and men more inclined towards loss of work capacity and income generation. Stigma was experienced in different social spaces: women described fertility-related and family-centered stigma, while men more often reported stigma within peer environments. It was also noted that both men and women reported concealing their diagnosis in certain contexts to avoid negative reactions. These patterns suggest that gender does not change the presence of diabetes stressors but shapes where and how they are experienced and managed across occupational, generational, and sociocultural domains [68, 69]. Similar patterns have been reported in previous studies, where women tend to emphasize relational and family consequences of diabetes, while men focus more on occupational functioning and productivity [70].

These findings advance understanding of the psychosocial burden of diabetes in Ghana’s Ashanti Region, addressing gaps in clinic-centric literature. The dual-site design revealed terrain-specific (Mampong) versus occupational (Obuasi) stress pathways, extending insights beyond single-center studies [71]. Age-disaggregated analysis uncovered generational differences: youth stigma versus elder complication anxiety, that were previously unexplored. Akan-specific taboos (shrine exclusion, food prohibitions) emerge as underexplored cultural mediators, paralleling global frameworks of menstrual stigma [72]. The structured use of FGD matrices and triangulation enhances analytic transparency and may provide a useful model for qualitative NCD research in similar African context.

### Limitations of the study

Participants were recruited from clinic registers at Mampong Municipal and Obuasi Government Hospitals, which may have resulted in the inclusion of individuals more engaged with formal healthcare services. It underrepresented undiagnosed individuals, treatment defaulters, and those outside formal clinics who may face greater distress, and limited transferability beyond Ashanti’s rural-peri-urban contexts.

Data were collected over a relatively short period (November-December 2025), and provided a snapshot of participants’ experiences. While this allowed in-depth exploration of stressors at a specific time point, it does not capture how diabetes-related stress and coping strategies may change over time or establish temporal relationships between stressors and quality-of-life outcomes.

We focused on patient (*n* = 92) and provider (*n* = 12) voices from mining- and farming-heavy areas, which may have limited inclusion of male family perspectives on household stigma and other occupational groups. The study may also be influenced by social desirability bias, recall bias, and observer effects during data collection. Logistical constraints, including rainy season access challenges, led to member checking being conducted during final FGDs rather than through iterative participant validation across multiple waves. We addressed this with ≥ 85% interrater reliability, peer debriefs, transcript audits (20% sample), and reflexivity.

Future studies should adopt longitudinal designs, include non-clinic samples (defaulters, households), and balance genders/occupations. This would deepen insights into diabetes distress amid Ghana’s agrarian-industrial stressors.

### Implications for policy and practice

These findings underscore the need to integrate psychosocial stress screening into the Ghana Health Service (GHS) non-communicable disease (NCD) protocols. Annual diabetes distress assessment should be conducted alongside routine HbA1c testing to identify patients at risk of stress-driven non-adherence.

Financial barriers were a primary driver of treatment default. Implementing medication affordability vouchers covering up to 75% of insulin and transport costs for patients spending more than 30% of their household income on diabetes care could directly address this challenge. This recommendation is supported by patterns observed across all 16 focus group discussions (FGDs), where cost-related non-adherence was universally reported.

Health system interventions should prioritize decentralization and occupational support. Establishing Community-based Health Planning and Services (CHPS) satellite clinics in hilly districts such as Mampong would mitigate transport-related barriers. In occupational settings, mining industry protocols mandating meal breaks for shift workers could prevent hypoglycaemia, as observed among Obuasi miners. Furthermore, training nurse specialist cadres in psychosocial first-line support would address the widespread shortage of health worker counseling, ensuring patients receive guidance on stress management, insulin use, and foot care.

Community and cultural interventions are equally important. Church-based diabetes fellowships that leverage existing spiritual coping networks could provide scalable peer support, with the potential to improve adherence, drawing on evidence from similar community-based HIV care models. In parallel, Akan traditional healer taskforces and spousal training modules could reduce taboo-driven treatment default and family stigma, which affected 13 of 16 FGDs in this study. These culturally tailored interventions complement clinical strategies and strengthen holistic diabetes management.

## Conclusion

This study shows that diabetes distress in Ghana’s Ashanti Region is shaped by where people live, the work they do, their age, and the cultural norms that surround diabetes. Transport barriers in Mampong, mining shift work in Obuasi, fear of complications among older adults, stigma among younger adults, and Akan taboos all combine to make daily diabetes management difficult.

Ghana’s NCD response must move beyond clinic-based care. Routine psychosocial screening should be part of diabetes care, alongside HbA1c testing. Insulin costs that consume 50–75% of household income must be reduced. Services should be brought closer to people through CHPS satellite clinics in hilly districts. Community systems also matter. Churches, families, and traditional healers should be part of diabetes support, not outside it. Together, these changes can reduce distress, improve adherence, and restore dignity for adults living with diabetes in peri-urban Ghana.

## Supplementary Information

Supplementary Material 1.

Supplementary Material 2.

## Data Availability

The semi-structured interview guides and topic facilitation tools used in this study are available as Supplementary Material 1. De-identified participant transcripts are not publicly available due to ethical restrictions protecting participant confidentiality. Substantial portions of the data are presented in the manuscript as anonymized verbatim quotations. Where additional access is required for legitimate research purposes, de-identified data may be made available upon reasonable request to the corresponding author (Mr. Godfred Darko), subject to ethical approval and confidentiality safeguards.
