# Supplementary material for: Living with type 2 diabetes: stress and daily life challenges among adults in the Ashanti Region, Ghana
Source: BMC Public Health. 2026 Jun 12;26:2390. doi: 10.1186/s12889-026-28131-4 (PMC13483566; doi:10.1186/s12889-026-28131-4)
Supplement: Supplementary file 1 [file 12889_2026_28131_MOESM1_ESM.docx]

**Supplemental Material 1: Semi-Structured Topic Guide**

### Title: Living with Type 2 Diabetes: Stress and Daily Life Challenges Among Adults in the Ashanti Region, Ghana

Study Reference: GHS-ERC: 053/09/25
Fieldwork Period: 22 November – 11 December 2025
Languages: English (primary); Asante Twi (for clarification only)

### Target Groups

### Focus Group Discussions (FGDs)

Participants: Adults with Type 2 diabetes

Number of groups: 16

Group size: 6–10 participants per group

Age categories: 30–45 years, > 45 years

**In-Depth Interviews (IDIs)**

Participants: Health workers

Total: 12

Cadres: Nurses, physicians, counselors

Session Duration**:** 45–60 minutes per session

**FGD GUIDE FOR DIABETIC PATIENTS**

**Introduction**: Good morning/afternoon. Thank you for coming. We are researching how stress related to diabetes affects daily life. Your experiences will help improve care services. Everything you share will remain confidential. You are free to leave at any time. May we record this discussion?

**SECTION 1: DAILY DIABETES EXPERIENCES (15 minutes)**

### Q1: Can you describe what a typical day looks like managing your diabetes?

**Probes:**

- When do you test your sugar levels? How often?
- What challenges do you face getting medications?
- How much time does diabetes management take daily?
- What feels most burdensome about daily care?

### Q2: What situations make you feel stressed about your diabetes?

**Probes:**

- Financial worries (medication costs, clinic transport)?
- Family reactions or support?
- Fear of complications (eyes, kidneys, amputations)?
- Missing work or social events?

**SECTION 2: QUALITY OF LIFE IMPACT (20 minutes)**

### Q3: How does diabetes stress affect your daily activities?

**Probes:**

- Work performance or farming/mining productivity?
- Time spent with family or children?
- Participation in church or community events?
- Sleep patterns or energy levels?

### Q4: Can you share a specific time when stress affected your health?

**Probes:**

- Did stress change your eating habits?
- Did you skip medications or clinic visits?
- Any hospital admissions triggered by stress?
- How did symptoms (pain, fatigue) worsen?

**SECTION 3: COPING AND SUPPORT (15 minutes)**

### Q5: What helps you cope when diabetes stress feels overwhelming?

**Probes:**

- Talking to family, friends, or pastors?
- Church prayers or spiritual support?
- Traditional healers or herbal remedies?
- Resting or changing daily routines?

### Q6: How do cultural beliefs influence your diabetes stress?

**Probes:**

- Do people say diabetes comes from witchcraft or sin?
- Are there restrictions during symptomatic periods?
- How do Akan traditions affect diabetes management?
- What do community members say about diabetes?

**CLOSING (5 minutes)**

### Q7: Is there anything else about diabetes stress we haven’t discussed?

Thank you for participating. Here is information about diabetes support groups and available financial assistance programs.

**IDI GUIDE FOR HEALTH WORKERS**

**Introduction:** Good morning/afternoon. Thank you for your time. We are documenting patient stress experiences to improve diabetes care. Your professional insights are important. All responses will remain confidential. May we record this interview?

**SECTION 1: OBSERVED STRESS PATTERNS (10 minutes)**

### Q1: What are the most common stress factors you observe among diabetic patients?

**Probes:**

- Financial barriers to treatment adherence?
- Family conflicts over diabetes management?
- Occupational challenges (farming, mining)?
- Most frequent emotional responses?

### Q2: How does patient stress typically affect their diabetes control?

**Probes:**

- Changes in HbA1c or blood glucose patterns?
- Increased clinic visits or hospitalizations?
- Treatment defaulting patterns?

**SECTION 2: QUALITY OF LIFE OBSERVATIONS (10 minutes)**

### Q3: What quality-of-life impacts do you notice from patient stress?

**Probes:**

- Work absenteeism rates?
- Family relationship breakdowns?
- Social withdrawal patterns?
- Most affected age groups or occupations?

**SECTION 3: CULTURAL AND COMMUNITY FACTORS (10 minutes)**

### Q4: How do cultural beliefs influence patient stress experiences?

**Probes:**

- Akan taboos or spiritual explanations?
- Community stigma effects on clinic attendance?
- Gender differences in stress expression?

### Q5: What coping strategies do patients find most helpful?

**Probes:**

- Faith-based coping effectiveness?
- Peer support group outcomes?
- Family involvement benefits/challenges?

**CLOSING (2 minutes)**

### Q6: What else should we know about diabetes stress in this community?

Thank you for your valuable insights.

## Language and Transcription Note

All interviews and focus group discussions were conducted in English using structured and semi-structured guides. Where participants required clarification, brief explanations were provided in Twi by trained bilingual facilitators to ensure comprehension without altering the meaning of questions.

Audio data were transcribed verbatim in English, with bilingual verification of clarification segments and key meaning units by independent Twi–English reviewers to ensure interpretive accuracy. No full Twi transcripts were produced.

**FACILITATOR NOTES**

**FGD Logistics**

- Groups stratified by age (30–45 years, >45 years) and gender where possible
- Venues: Hospital conference rooms or community centers
- Refreshments provided per cultural norms
- Facilitators: 1 Lead facilitator (questioning), 1 Note-taker (non-verbal observations)

### Quality Control

- Audio recordings and handwritten field notes
- Immediate post-session debriefing
- Member checking: summary of key points at session end
- Saturation monitoring: no new themes after 12 FGDs

### Ethical Protocols

- Verbal and written consent (witnessed for low-literacy participants)
- Right to withdraw at any time
- No personal identifiers recorded
- Pseudonyms used in all quotations
- Distress protocol: referral to counseling services when needed
