# Supplementary material for: Living with type 2 diabetes: stress and daily life challenges among adults in the Ashanti Region, Ghana
Source: BMC Public Health. 2026 Jun 12;26:2390. doi: 10.1186/s12889-026-28131-4 (PMC13483566; doi:10.1186/s12889-026-28131-4)
Supplement: Supplementary file 2 [file 12889_2026_28131_MOESM2_ESM.docx]

## COREQ (Consolidated Criteria for Reporting Qualitative Research) Checklist

**Manuscript:** Living with Type 2 Diabetes: Stress and Daily Life Challenges Among Adults in the Ashanti Region, Ghana

## Domain 1: Research Team and Reflexivity

1. **Interviewer/facilitator:** FGDs and IDIs were facilitated by Darko Godfred and Georgina Anokye Agyekum.

- *Page:* Methodology – Data Collection

2. **Credentials:** D.G. - MPhil Public Health; G.A.A. - BSc Nursing

- *Page:* Title Page

3. **Occupation:** Teaching Assistants/researchers (Public Health Education; Nursing)

- *Page:* Author affiliations (Title page)

4. **Gender:** Mixed gender research team

- *Page:* Data collection

5. **Experience and training:** Trained qualitative researchers; bilingual facilitators fluent in Asante Twi and English; trained note-takers

- *Page:* Data Collection

6. **Relationship established prior to study:** No prior relationship established

- *Page:* Data Collection / Ethics

7. **Participant knowledge of interviewer:** Participants informed of academic purpose and health research objectives

- *Page:* Ethical Considerations

8. **Interviewer characteristics:** Public health focus, diabetes research interest, culturally embedded bilingual facilitators

- *Page:* Data Collection / Analysis

**Domain 2: Study Design**

9. **Methodological orientation and theory:** Qualitative descriptive design; content analysis

- *Page:* Study Design and Sampling

10. **Sampling:** Purposive sampling

- *Page:* Study Design and Sampling

11. **Method of approach:** Face-to-face recruitment via clinic referrals

- *Page:* Data Collection

12. **Sample size:** 92 patients (FGDs) and 12 health workers (IDIs) = 104 participants

- *Page:* Methods / Results - Study Participants

13. **Non-participation:** All participants participated fully

- *Page:* Data Collection

14. **Setting of data collection:** Clinics and community settings in Mampong Ashanti and Obuasi Municipal

- *Page:* Study Area / Data Collection

15. **Presence of non-participants:** All invited participants agreed to participate; no refusals were reported

- *Page:* Data collection

16. **Description of sample:** Adults with T2DM, age groups 30–45 and >45 years; nurses, physicians, counsellors; data collected Nov–Dec 2025

- *Page:* Study Participants / Data Collection

17. **Interview guide:** Semi-structured interview guide developed in English, administered in English with real-time interpretation into local Asante Twi during FGDs/IDIs where required (Supplementary Material 1)

- *Page:* Data Collection

18. **Repeat interviews:** No repeat interviews

- *Page:* N/A

19. **Audio/visual recording:** Audio recording used

- *Page:* Data Collection / Data Management

20. **Field notes:** Field notes taken during and after FGDs/IDIs

- *Page:* Data Collection / Data Management

21. **Duration:** 45–60 minutes per FGD/IDI

- *Page:* Data Collection

22. **Data saturation:** Achieved by 12th FGD

- *Page:* Results / Triangulation Matrix

23. **Transcripts returned:** Member checking conducted through summary validation (not full transcript return)

- *Page:* Data Analysis

**Domain 3: Analysis and Findings**

24. **Number of data coders:** Two independent coders

- *Page:* Data Management and Analysis

25. **Description of coding tree:** Thematic structure and subthemes described

- *Page:* Data Management and Analysis / Results – Thematic Structure

26. **Derivation of themes:** Both deductive (guide-based) and inductive (emergent)

- *Page:* Data Management and Analysis

27. **Software:** Data management and coding conducted using NVivo 14, with supplementary analysis in Excel

- *Page:* Data Management and Analysis

28. **Participant checking:** Member checking through summary validation

- *Page:* Data Analysis

29. **Quotations presented:** Yes

- *Page:* Results – Themes 1–5

30. **Quotation identification:** Yes (location, age group, role, IDI/FGD)

- *Page:* Results

31. **Consistency between data and findings:** Yes

- *Page:* Results / Discussion

32. **Clarity of major and minor themes:** Major themes clearly presented; minor/diverse cases described in subthemes

- *Page:* Results – Thematic Structure
